# Promoting Personal Growth through Experiential Learning: The Case of Expressive Arts Therapy for Lecturers in Thailand

**DOI:** 10.3389/fpsyg.2017.02276

**Published:** 2018-02-05

**Authors:** Bussakorn Binson, Rachel Lev-Wiesel

**Affiliations:** ^1^Center of Excellence in Thai Music & Culture Research, Faculty of Fine and Applied Arts, Chulalongkorn University, Bangkok, Thailand; ^2^The Emili Sagol CAT Research Center, University of Haifa, Haifa, Israel

**Keywords:** experiential learning, expressive arts therapies, personal growth, self-figure drawing, professionals

## Abstract

The aim of the paper is to assess academic experiential learning in relation to academic lectures' perceived personal and professional growth. Sixteen PhD students (age ranged between 23 and 46, 10 male, 6 females) participated in an introduction to expressive art therapy. Qualitative methods according to phenomenological methodology was used. At the beginning and end of the 48-h course they were asked to draw themselves, and explain the differences between the two drawings. In addition participants were semi-structured interviewed about the course and its personal and professional aspects at the end of the course. The main themes were the carousal of emotional experience, the use of art means for growth, and, professional growth. Findings revealed a perceived growth in terms of family relationships, inter—personal skills, and professional role performance.

## Introduction

Experiential learning is a philosophy and methodology in which educators purposefully engage the students in experiences and focused reflection in order to increase knowledge, develop skills, and clarify values (Association for Experiential Education, para. 2). The Experiential Learning Theory developed by Kolb (1984) provides a holistic model of the learning process and a multilinear model of adult development. It emphasizes the central role that the person's subjective experience plays in their learning process. Learning from both cognitive and emotional perspectives is at the heart of experiential learning (Davis, 2011).

Experiential learning is a form of practice-based education that provides exposure and opportunities for students to explore interpersonal dynamics during work, along with the roles and identities they will encounter as future professionals (Wheeler and Grocke, 2001). There is a general agreement across disciplines that educational programs for future therapists should include both didactic and experiential components since learning involves both cognitive and affective processes (Dudley et al., 1998). Thus, learning is based on active personal experience in combination with theoretical concepts. It is through active participation in a learning process that students acquire interpersonal skills, develop an understanding of the therapeutic process, and increase their self-knowledge. Additionally, experiential learning encourages students to recognize and reflect upon their own interpersonal style and to identify areas which need to be developed (Hall et al., 1997), both as individual human beings and as future therapists. For example, students in expressive arts therapies would draw their safe place as humans and safe place as therapists in a therapeutic encounter. Finding its meaning and discussing it within the class allows them to review it from different aspects; personal and professional, individual and group member, etc.

Despite the broad use of experiential learning components in academic therapy education, the empirical evidence on its impact in terms of professional growth is scarce due to the unique problem of assessment (Qualters, 2010) and the variety of exercises employed in different therapeutic disciplines (i.e., psychology, social work, art therapy, or occupational therapy). Professional growth refers to the development of interpersonal skills–that help professionals in creating relationships with clients (Muran and Barber, 2011). These skills as self-awareness, empathy, warmth, and congruence, are known to relate to one's own mind and others well-being, both on a professional and personal level (Elman et al., 2005). In relation to Thai culture, professional growth means broadening the practitioner's knowledge on one hand, and deepens the understanding for human sufferings. Experiential activities in academic education consist of the means as well as the ends; it is imperative therefore to look at assessment as more than outcome measurement. Wurdinger (2005) asserted that development of innovative tools measuring both the process and the product are imperative in order to assess students' professional and personal growth. For example, Knecht-Sabres (2013) found recently that experiential learning (EL) in occupational therapy academic studies, is an effective method to enhance the understanding and application of course material, improve the personal and professional attributes and skills needed to be an effective clinician, and to improve clinical reasoning skills.

Despite the general consensus that EL is an important element in academic therapy studies together with its vast use within theoretical courses as a supplement, the evidence on its impact mainly relates only to its implementation within field studies, practicums and supervision (e.g., Schreiber et al., 2015). In light of the scarcity of evidence on EL and its perceived impact on academic studies related to therapeutic training, the current study attempted to evaluate the impact and significance of experiential learning in an expressive art therapies course of Thai PhD students. The main research question was: how does EL in expressive art therapies education contribute to self-awareness and growth of students? More specifically, the study sought to find out the impact of experiencing expressive art means on personal and professional levels of PhD Thai students. It was hypothesized that arts based experiential learning, followed by therapeutic sharing amongst classmates, would impact each participant's growth that would be reflected in self-awareness both personally and professionally. It was also hypothesized that EL would contribute to the group cohesiveness.

### Experiential learning and expressive arts therapies

The expressive arts therapies is an hybrid therapeutic profession aiming toward a better integration between body and mind (Lev-Wiesel, 2015). According to Lusebrink et al. (2012) expressive arts therapies consists of three stepwise levels—Kinaesthetic/Sensory, Perceptual/Affective and Cognitive/Symbolic—interconnected by the creative level. Thus, the creative therapeutic process engages physiological sensations, emotions, and cognitions; verbal and non-verbal narration and expressions, for improving people's psychological and social well-being. Art making consists of creation, observation, reflection, meaning making and insight that may lead to change (Malchiodi, 2005). McNiff (1981) asserted that expressive therapies are those that introduce action to psychotherapy and that action within therapy is imperative since life is rarely limited to a specific mode of expression.

The rational for including experiential learning within the majority of expressive art therapy academic courses is the belief that education of expressive art therapies has to be a place where both cognitive and emotional material come together to allow the future practitioners to reflect about themselves and their clinical work on the conscious level (Mollon, 2000). Thus, many of the theoretical courses within these programs provide students a space for thinking, feeling, and reflecting.

The benefits of experiential learning through the use of the expressive arts include within academic programs: improving self-awareness, developing conceptualization skills, and processing countertransference (Turry, 2001). Some other benefits include: self-care and stress reduction (Deaver and Shiflett, 2011), an improved supervisory relationship (Scheiby, 2001), self-awareness on the part of the student (Austin and Dvorkin, 2001), empathic attunement (Cooper, 2001), and a sense of empowerment as a future therapist (Proctor et al., 2008). For example, Ko (2014), who examined the experience of six native Korean expressive arts therapy students (four in art therapy and two in dance/movement therapy) in a movement-based program at a Korean university, reported that there was a reduction in perceived authoritarianism paired with an enhancement of verbal sharing with their clinical supervisor. Another recent study conducted by Elkis-Abuhoff et al. (2011) that focused on the development of professional identity in creative art therapy academic setting, found an increase of the importance of self-awareness, supervisory relationships and professional identity, at the completion of the program. Consistent with the previous study, Deaver and Shiflett (2011) reported that using art techniques within expressive art therapies educational field studies was effective in reducing supervisee stress, which in turn increased supervisees' self-awareness, allowed for better case understanding and intervention conceptualization along with the processing of countertransference issues. However, in light of the scarcity of evidence on the impact of experiential learning on expressive art therapies' students, the current study focused on the personal and professional growth experienced by students who participated in an academic course entitled Introduction to Expressive Art Therapies at Thailand's Chulalongkorn University. This course was conducted by a Thai female professor who is an expert in Thai music and imagery therapy and an Israeli female professor who is an expert in expressive art therapies.

## Methods

The present article aims to contribute an insider's view of the learning experience of expressive art therapies in Thai doctorate students. The descriptive phenomenological method guides our methodology (Giorgi, 2012). This perspective allows us to capture lived experience and to conceptualize it, offering insights into how individuals in particular context (course within their academic studies) make sense of a given phenomenon (the course characteristics) (Giorgi, 2012).

### Participants and procedures

Following approval of CU Institutional Review Board (IRB) and signing on a consent form to participate in this research (participants gave permission to use their names on the presented drawings, as well as signed a consent form to use and publish the photo in Figure 4), 16 Thai PhD students were registered to an elective PhD course in expressive arts therapies. The course syllabi included information about the course and the study objectives. All of the students (10 males and 6 females, born and raised in Thailand) had MAs in the arts (visual arts, creative arts, dance or music) and already were in positions as lecturers at different academic institutions located in the greater Bangkok area. The course was the first encounter between the students and the lecturers who had extensive experience and knowledge in expressive arts therapies. The participants' ages ranged from 24 to 43 (see Table 1 for participants' demographics). The participants used more than one art medium in their classroom instruction beyond their own art medium, but none of them had experience in using media of art for therapeutic purposes.

**Table 1:** Participants' demographics.

**Participant no**.	**Major subject**	**Gender**	**Marital status**	**Age**
1	Creative arts	F	S	34
2	Dance	M	S	35
3	Visual arts	F	S	26
4	Creative arts	F	S	43
5	Dance	M	S	31
6	Music	M	S	40
7	Music	F	M	36
8	Creative arts	M	S	33
9	Creative arts	F	M	38
10	Creative arts	M	S	34
11	Visual arts	M	S	43
12	Music	F	M	33
13	Visual arts	M	S	33
14	Creative arts	M	M	34
15	Creative arts	M	S	29
16	Creative arts	M	M	31

The 48-h course included 16 three sessions during the first semester of 2015 academic year. The course covered the theoretical background of expressive art therapies (e.g., art as therapy vs. therapy as art), basic therapeutic concepts (e.g., containment, transference and counter-transference, and corrective experience/reparation, etc.), and intervention techniques (e.g., drawings for diagnostic and therapeutic purposes, the use of visual arts or movement and dance therapy in treating sexually abused children). The students needed to select a concept and a population group to study in-depth, develop an intervention technique and make a 20 min classroom presentation followed by a session of implementing their intervention on their classmates (see Table 2 for a detailed program). These sessions drawn by the participants included a concrete experience-engagement, a reflective self-observation noticing what happened and its relation to one's life, and summary of conceptual understandings by the student conductor and lecturers of the course.

**Table 2:** The “introduction to EAT” course program.

**Session No**.	**Content**	**Technique used**
1.	Introduction and Warming Up	History of EAT (Ice breaking and being a human) Example for exercise: Know your classmate by name through acting and movement (personal meaning to each name based on personal history, color and attached movement)
2.	Introduction to EAT	Theoretical background of expressive art therapy Example for exercise: Draw your safe place, sharing with classmates
3.	Use of drawings for diagnostic purposes	Drawing tests, DAP, HTP Example for exercise: Draw yourself, draw a house, tree, and person of the other sex Add narratives; imitate the posture of the figure drawn, share its feelings and thoughts; Analyze in pairs according instructions
4.	Music Therapy	Theoretical background of Music therapy Example for exercise: Guided Imagery Music (listening to music working in a pair, then write the images of partner, sharing). Group sharing of feelings and sensations
5.	Use of drawings for therapeutic purposes	Theoretical framework Trauma and child abuse Example for exercise: trauma and post-traumatic growth– “Draw an uneasy event in your life”, upon completion, “copy it as accurate as possible and add to it what had helped you cope”; Sharing, interpreting of drawings and discussion within the group
6.	Psychodrama	Theoretical background Example for exercise: Draw your family in an activity; The protagonist set the pictorial scene and works it through. Sharing within the group at the closure
7.	Use and meaning of colors in EAT	Theoretical background Example for exercise: The self-revelation technique through colors technique (Lev-Wiesel Daphna-Tekoha, 2000)
8.	Dance and movement therapy	Theoretical background Example for exercise: Comforting vs. ritual movement in trauma exercise
9.	Play and drama Therapy	Theoretical background Example for exercise: Improvisation in movement, drama and play - Anger expressions—anger management - Listening to oneself and others' heart beating; synchronizing between the heart beatings - Group hug
10.	Movement therapy and Trauma	The body keeps the score- theoretical model Example for exercise: Release Exercises 6 exercises to stimulate tremor in order to release trauma/tension
11.	Research presentation 1 (7 m/p) Student presents an updated empirical research in EAT within the classroom	Group discussion and sharing
12.	Research presentation 2 (7 m/p) Student presents an updated empirical research in EAT within the classroom	Group discussion
13.	Students presentation of their own creative technique 1 (25 m/p) Each student presents their own creative technique using the classmates	Group sharing and discussion
14.	Students presentation of their own creative technique Students presentation of their own creative technique 1 (25 m/p) Each student presents their own creative technique using the classmates	Group sharing and discussion
15.	Students presentation of their own creative technique 1 (25 m/p) Each student presents their own creative technique using the classmates	Group sharing and discussion
16.	Closure	Example for exercise: Draw yourself Comparison with the self-figure drawn prior to beginning of the course in terms of feelings and well-being; questions unanswered; closing ending summary and comments; group departure.

### Procedures and measurements

The study employed qualitative methodology—self-figure drawing and semi-structured open-ended questionnaire as well as written protocols of the class sessions taken by the research assistant. The students were asked to draw themselves both before and after the end of the course (pre-post) and complete a semi-structured interview (post-only).

The rational for using qualitative methods (self-figure drawings and semi-structured interview) was based on a transformative paradigm (Evans et al., 2008) that asserts the value of promoting group and individual empowerment and change (Ozanne and Saatcioglu, 2008). These transformations occur across the individual and the group to which he or she belongs, through a learning process occurring concurrently between the researchers (the lecturers in the current study) and the group participants through challenging activities and actions (Perkins et al., 2007). This transformation relates to learning as a form of personal and professional research that also implicates an individual's affiliation to a particular group. As a result, transformative change modifies the problems experienced by individuals to equip them with the ability and belief that they can navigate and acquire the resources needed to improve their quality of life (Bandura, 2004) and in this case, the quality of their lecturing performance. Thus, the goal of research within a transformative paradigm is to move individuals from the margins and toward personal and/or social change.

### Semi-structured interview

The semi-structured interview was guided by descriptive phenomenological-psychological perspective. This view offers to provide the lived experiences of participants and understand them, allowing insights into how individuals in particular contexts make sense of a given phenomenon (Denzin and Lincoln, 2005; Spinelli, 2005). Phenomenology is an intense examination of individual experience. It is an embodied examination of perspective and meaning (Sokolowski, 2000; Willis, 2007).

Thus, the interview included the following issues:

- Pre (retrospective)-and post-perception of the course
- Quality of Interpersonal relationship in the group
- Role of the lecturer
- Comfort level
- Level of verbal sharing
- Benefits or deficiencies regarding the use of experiential learning

### Self-figure drawings

This study's instruction to “Draw yourself” is based on the Draw-A-Person Test (DAP), developed by Machover (1949). It is based on the concept that the figure drawn represents the drawer, while the paper represents the drawer's environment. According to Furth (2002), Gillespie (1994), and Lev-Wiesel (1999) and Lev-Wiesel and Hershkovitz (2000) the figure drawn usually reflects the drawer's deep acquaintance and inner knowledge of oneself. Klepsch and Logie (1982), noted that drawings represent what a person is like on the day he or she produces the drawing. Keeping that caveat in mind, the analysis should consider the overall impression of the picture. Some overall impression descriptive word pairs are happy/sad, friendly/unfriendly, active/passive, and strong/weak. This general impression provides a clue to the drawer's mood at the time the picture was drawn. Additionally, the main themes emerging from the picture should be found. If the same theme is apparent within the drawing, such as sadness, it provides a stronger indication to the drawer's state of mind.

Other aspects of the drawing to consider in forming an overall impression are the size of the figure, its placement on the page (indicative of feelings of inferiority, inadequacy, and insecurity), facial expressions (indicative of self-esteem, state of mind), body posture (indicative of self-confidence) and shadowing or omission of body parts (indicative of anxiety and of problems relating to others) (Furth, 2002; Carmaichael, 2006). Furthermore, based on the supported assumption that cultural values are represented in self-figure drawings (Rubeling et al., 2010) and taking into account Thai cultural values—kindness is represented by a smiling face, while modesty and cleanness is represented by the omission of one's feet (Komin, 1990). The two following indicators were selected for comparison in this study: facial expressions and the omission of the lower part of the body. Based on previous studies indicating that (1) happiness is the most universal facial expression (smile with displaying teeth and crescent-shaped eye) have the same meaning across all cultures (Collins, 2003), (2) a smile indicates health social invitation, strength, and self-sufficiency (e.g., Remland and Jones, 2005), (3) drawing the whole body and placing it in the center of the page mean self-confidence (Koppitz, 1968; Lev-Wiesel and Drori, 2000), it was hypothesized that the personal growth through experiential learning is likely to be presented in happier facial expressions and a safe body position of the whole figure (including the feet) placed in the middle part of the paper.

Participants were asked to draw themselves on a sheet of A3 paper with a pen or pencil at the beginning of the course, and again at the end of the course. Any questions the students asked regarding how to proceed with the drawing were answered by the instructor with the phrase “As you wish.” When completed, the participants were asked to provide a verbal narrative for the drawing to the group. On the final day of the course, after their last drawing, the participants were given their first pre-course drawings and were asked to describe the differences between the two. Their responses were recorded, transcribed and translated into English by a Thai-English translator.

#### Analysis

Data analysis included open coding to enable the identification of units of meaning. A cross-case analysis followed in which segments from each interview were condensed until core themes emerged (Patton, 2002). The themes, which emerged from the analysis, are differentiated from the categories of the interview guide. Each theme consisted of different categories that related to the following dimensions: personal space, comfort, relationship, therapeutic component, non-verbal expressive tools, professional development, and personal and professional awareness. Our cross-case analysis revealed that all those different perspectives fall under a unified spectrum ranging from communication as a tool to gain and provide support to communication between their social roles; personal and professional. Three primary themes emerged from the data and serve as the backbone of our analysis.

#### Trustworthiness

In the present study, credibility is achieved through the systematic presentation of quotes and the analyses, which allow the reader to evaluate the ways in which reality was constructed and themes were derived (Henwood and Pidgeon, 1992; Maxwell, 2005). In qualitative research, the emphasis shifts from validity to validation. Rather than presenting a finished product, researchers describe the process by which they arrived at the specific constructions underlying the study and thus allow the readers to make their own judgments and to validate or reject the interpretations suggested (Patton, 2002). For instance, the theme Emotional experience is presented in the following manner: First, information regarding the group activities was offered, followed by quotes of participants. Afterwards, a more detailed description of the group's process is offered for the reader. This served as the validation tool for the researchers' systematic work. The focus in such research is on in-depth subjective analysis of experiences rather than on generalizations. Additionally, trustworthiness is also ensured by collecting various types of data (i.e., semi-structured interview, self-figure drawings at the beginning and ending of the course, and participants interviews) (Stake, 2010; Creswell, 2013). Important to note that the two sets of drawings (pre and post) were given to two practitioners (a dance and movement MA therapist and an MA art therapist) who were asked to estimate the following indicators hypothesized to represent self-awareness, depression, anxiety, and self-control: body shape—omission/whole body (head only, head and torso, whole body); facial expression (sad, smiling, or detached); and placement of the body on the paper (on top, medium or lower part of the paper). Note that pre- and post-drawings do not represent different constructs, thus all 32 drawings were rated accordingly and the ratings went into the same reliability test. This procedure is carried out in order to determine the indicators for comparison. Since there were only two raters, the final score (correlation) was determined by averaging the two assessments. Inter-evaluator reliability was 0.87 (Spearman correlation).

## Results

The 16 self-report semi-structured interviews yielded three themes: (a) the emotional carousal experience, (b) the use of art means for growth, and (c) professional growth, from seven categories (that appeared differently but in relation to each of the themes): personal space, comfort, relationship, therapeutic component, non-verbal expressive tools, professional development, and personal and professional awareness. Note, that an example for how the theme evolved of the categories mentioned above is presented in the first theme.

### Theme 1: the emotional carousal experience

Most participants reported having uncomfortable emotional experiences, such as fear, anxiety, and embarrassment (category- level of comfort) at the beginning of the first two sessions. This was mainly due to exercises that appeared to challenge their physical, emotional, and sexual boundaries (category- personal space). For example, in one of the exercises they were asked to listen to first their own and to another's heart beating by placing one ear to their back. Some of them felt uncomfortable in resting ear and head on another classmate (category- personal space/relationship). Some explanations for this unease were: the member was of the other sex, age/position disparity, and a general sense of invading the other's private space (category- personal and professional awareness). Following the lecturer's instruction of getting out of the classroom and return as merely a human being and prior to any gender or position or age distinction, enabled them to push themselves beyond the usual Thai cultural boundaries. For example, a female participant described “At the beginning I was really embarrassed, I even felt a bit paralyzed, I could hardly look to my fellows' eyes…I trusted the teacher and knew I need to comply. I watched my classmates and saw they overcome their embarrassment…after the second session, I became intrigued, wanted to experience the next session, wondered how I will feel…I was more and more excited, longed to come to the class, meet my friends, feel the warmth, hugs, sometimes cry because of the pain and suffering…felt so connected to others as if they were my family…”

One participant responded within the class: “I feel more as a human being, I feel I am a better—more forgiving person,” another male said “through mirroring and reflecting, each one's emotions were recognized,” (category- personal and professional awareness) another participant, female when relating to her experience wrote: “the important thing is to exchange my experience with friends and listen to their experiences, it fills me with love and understanding for others…being able to actually hear their hearts, gave me also an access to their souls (category- therapeutic component)…most importantly, I felt encouraged to help my fellow colleagues” (category- relationship/professional development).

All participants cited how profound this new, unfamiliar tool of using art as powerful means for self-exploration was through the enabling emotional expression, which resulted in a deep sharing through the disclosure of personal feelings and personal hardships. One participant whose family lost their house and property during the flood described “it was the first time I shared this event and how I feel with others. Crying in front of my classmates, disclosing my sorrow and pain with them, was unique experience for me…I moved from agony to inner calmness, can't explain it…it was like cleaning myself from within…” In addition, they found that while learning in a less structured format/manner, the emotional distance between the lecturers and students was decreased. Moreover, this shared transformative experience created a strong level of camaraderie through the sharing of both stories from the participants' life and the expression of a variety of emotions that most did not want to end.

The desire to continue spending time together after formal class hours by going together to a café or taking a trip together during the semester break was expressed by many. One male participant wrote: “I was happy every time I came to the class, I fear that after this course ends, all the opportunities to meet each other will be gone. It saddens me, because we became brothers.” A female participant stated: “I came early every time to the class to meet my colleagues. It was a happy time. We love and understand each other. We somehow liberated ourselves within the group. I wanted to tell the teachers I love them, I wanted to hug them each meeting. I have never hugged or been hugged before this course. At the last meeting, I hugged the teachers and my new friends, with tears of happiness dropping from my eyes.”

As can be seen, participants experienced a spectrum of negative and positive emotions and sensations (e.g., embarrassment, uneasiness, shame, joy, excitement, longing, freedom, happiness). These feelings were not only expressed toward their classmates but also to their family members (parents).

### Theme 2: the use of art means for growth

The use of art such as drawing can serve as a non-threatening gateway for students to verbalize their represented experiences and feelings, and convey their body's reactions during prior difficult and stressful events. Also, the development of metaphoric drawings and narratives can lead to a more conscious reflection and insights about themselves and those with whom they interact. Additionally, the meaning of their creation when shared within the group, whether it be drawing, sculpting, or dance and movement, tend to enhance the students' abilities to gain insight into transference and countertransference and the functional meaning of a therapeutic relationship. Namely, to experience a catharsis catalyzed from being creative and by receiving rich, in-depth feedback from the group's members on it. This feedback in turn, appeared useful in reaching conceptualization as reflected in the students' descriptions of the benefits of using art: ”I understood that crying is not a weakness it's just a means to show how one feels at the present. A smile on the other hand does not always represent happiness, one can hide behind it. I felt that by making related artwork and sharing it with others so they understand its meaning, the barriers between us disappeared.” Another participant mentioned: “As one of the professors of the course asked me when looking at my drawing 'are you a perfectionist? Does it interfere with your ability to relax?', “Yes,” I answered. That sentence was a very important reflection; it helped me become aware of its (perfectionism's) impact on my well-being.” Another participant described what the art making meant for him “it was like a revelation for me. I always tried to draw as I was taught, tried to make it better according my teacher's instruction…drawing how I feel, expressing what I had experienced, what helped me cope with the trauma (the flood left his family homeless), enabled me to see that I have resources…that I am strong…survived…I am blessed, no one died…”

The art creation became significant in learning about oneself, enabled participants to observe themselves through a distant, and acquire additional way to express their inner conflicts and difficulties to others.

### Theme 3: professional growth

All participants identified their areas of growth as human beings, instructors, and therapists. They reported a reduction in their expression of criticism toward others especially students, in addition to more sharing and self-disclosure, as the main areas of positive personal change. As one participant wrote: “I realized that people/students are just humans. My family members are just people who sometimes have good and bad days—as I do. My relationship with my girlfriend is much better now, we understand each other better. I used to criticize my students for everything, was very hard on them, kept distant. I feel I am a better teacher today, a teacher for life, an educator with empathy and understanding of life adversities, not an authoritarian lecturer.”

Professionally speaking therapists reported an attitude shift in their level of patience and openness toward creativity. They stated they are more bodily self-aware, have more emotional flexibility and are more comfortable with the use of the arts in sessions. For example, one female participant expressed: “I feel I have better access to other people's minds. More importantly, I felt encouraged in helping others. I am able to contain more suffering and relate to it without becoming intimidated or helpless.”

Instructors also reported a shift toward a mentor with equality and acceptance independent of position and status. They moved forward with greater awareness of student's emotional state and accompanying needs. One male instructor stated: “The teachers need to recognize themselves and their students as just people with stresses and address it…teaching my students that I am a person and a human being as they are, that we all have disadvantages and deficiencies, that we all can try to do our best and cam improve ourselves…this is what I realized and try to teach” Another said: “I feel students are humans like me. I will use the principles of art therapy within my class to help my students better understand themselves and feel reassured with their abilities.” Another wrote: “Learning by doing is the best way to learn. I understand and will do my best to better understand my students, to teach and be taught by them.”

#### Self-figure drawings

Frequency distribution revealed differences between the drawings in the following indicators: whole vs. omission of body (whole body 33% at the beginning, 80% at the end), facial expression (60% sad or detached at the beginning, 86% happy at the end), placement and posture of figure on the paper (100% were placed in the middle part of the page at the beginning, whereas 90% were standing firm on their feet, at the end).

Following observation of the two sets of drawings at the concluding session, each student commented on the differences between the drawings and their meaning. All participants shared their notions of their own personal growth in terms of confidence, trust in others, ability to share positive and negative feelings and traumatic events with friends and family members, ability to touch and perform gestures such as patting, caressing and hugging to reveal closeness with others, and strengthened abilities of decision making and problem solving. In addition, the drawings enabled the participants to evaluate their own performance before the activities began and after the course ended in terms of strengths, improvements, and insights.

**Figure 1:**
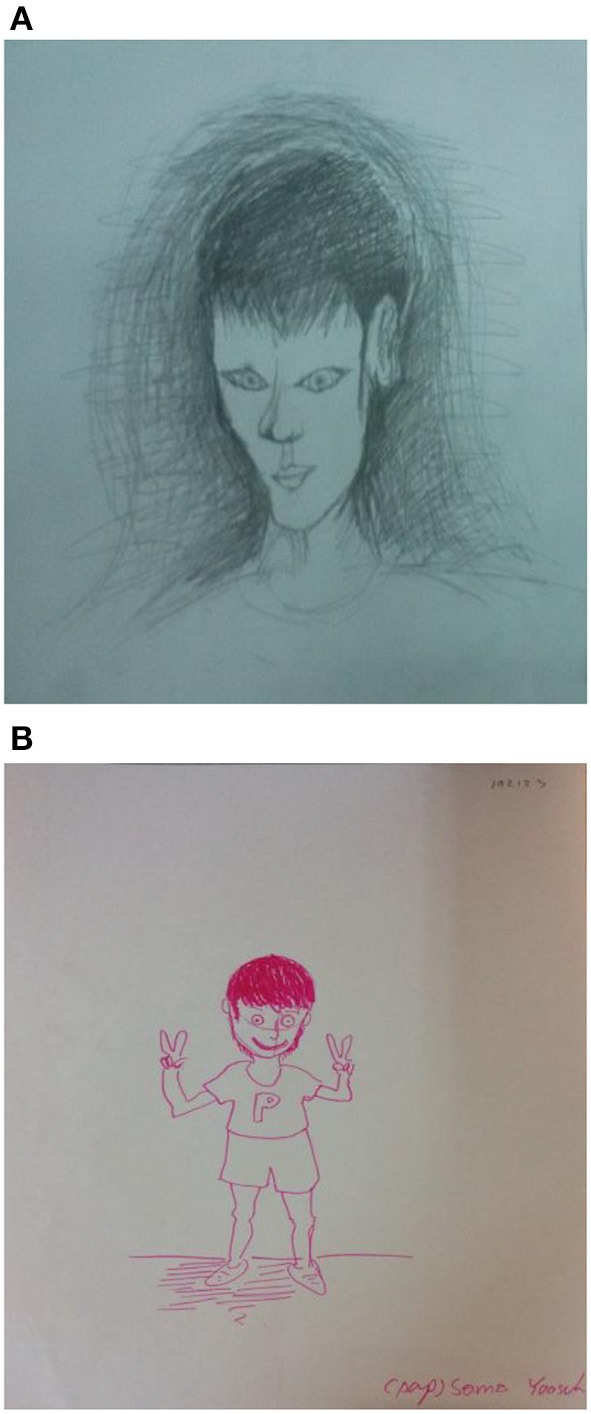
**(A)** The self-figure drawing of a male (aged 38) at the beginning of the course. **(B)** The self-figure drawing of a male (aged 38) at the end of the course.

**Figure 2:**
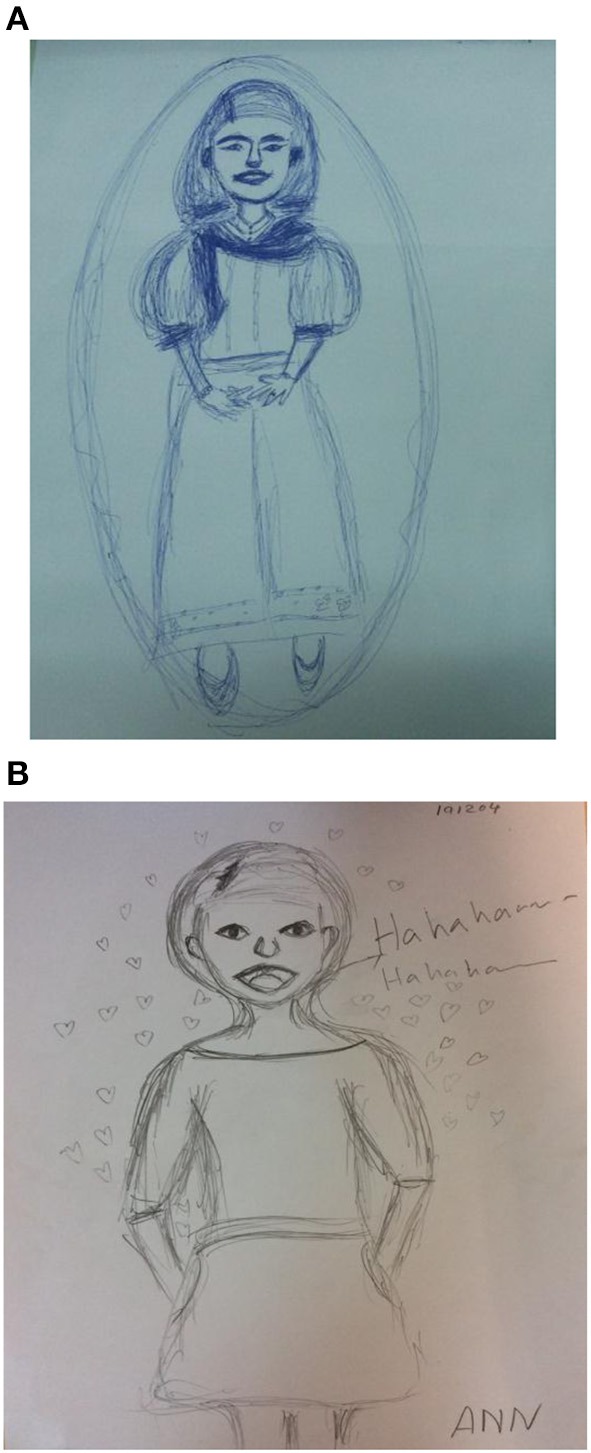
**(A)** The self-figure drawing of a female (aged 34) at the beginning of the course. **(B)** The self-figure drawing of a female (aged 34) at the end of the course.

**Figure 3:**
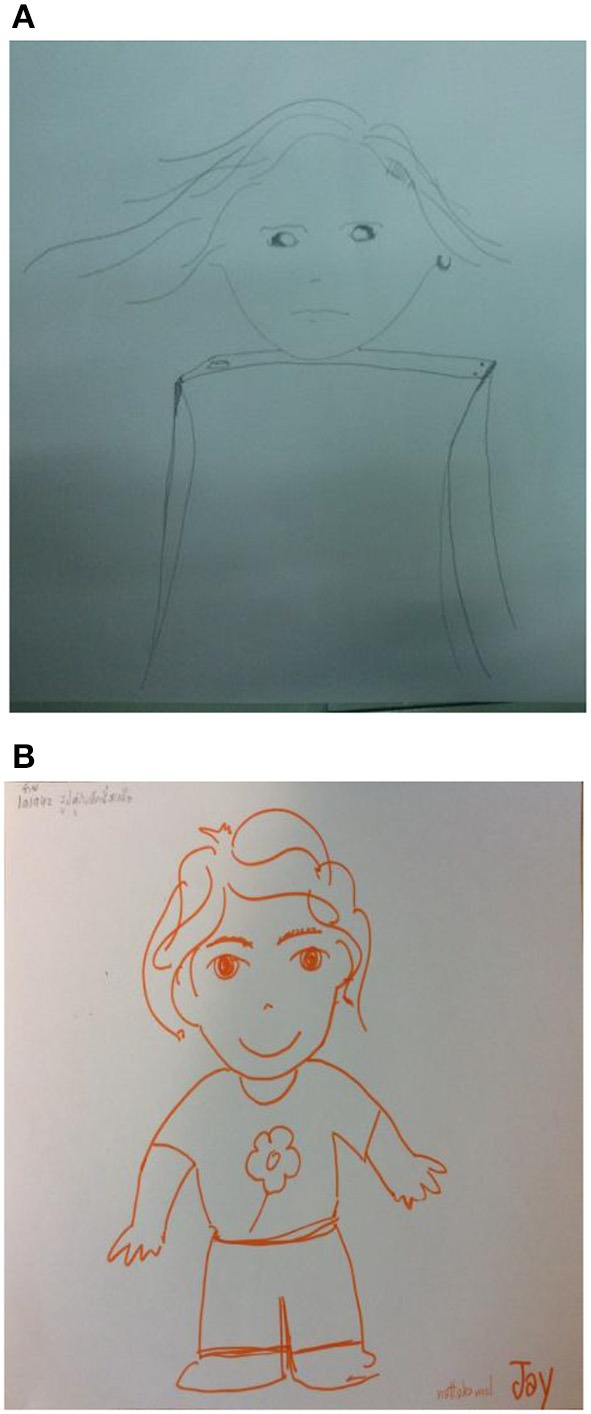
**(A)** The self-figure drawings by a female (aged 43) at the beginning of the course. **(B)** The self-figure drawings by a female (aged 43) at the end of the course.

The following are three examples:

Example 1
This first example is a male, aged 38, living with his girlfriend (see Figure 1). He shared his depression diagnose and suicidal thoughts with the group during the initial sessions. He expressed that the course's exercises enabled him and others to hug, share, and disclose feelings. The group's relationships which spontaneously extended beyond the group meetings helped lower his feeling of loneliness, which in turn helped him share his virtues as a talented musician and artist with his new friends. He became more aware of his influence on others including in the relationship with his partner. Looking at the two drawings he said: “I have learned that life can be good and enjoyable.” When viewing the pre- and post-course drawings, it is obvious that the drawer's feeling about himself and life are now totally different. Wherein the first drawn figure looks in agony: looking down, the head and face are shadowed all around, and the rest of his body omitted, the second drawn figure stands on its feet, smiling (e.g., The mouth can reveal happiness or sadness with a corresponding smile or frown, Koppitz, 1968). When turning to the post-course image with its raised fingers in a gesture of victory he said: “I have found friends, I became more aware of myself, I understood how much being depressed has impacted my relationship with my girlfriend, I feel I have gained my life back again.”
Example 2
In this example we have a female aged 34 who is a married music lecturer residing with her parents (see Figure 2). She disclosed during the course a history of social rejection by peers. She also expressed her ongoing fear of being rejected and under-valued by her colleagues despite her making enormous efforts at work both as a teacher and as a colleague. In her response to her first figure drawing, she said that she encapsulated herself for protection. It also enabled her to keep her own anger and rage toward others who hurt her contained. The second, or post-figure, reveals the beginning of coming out of her protective bubble. In reference to her experience in the class, she said that one of the most meaningful activities for her was hitting a pillow as it allowed her to vent her anger in front colleagues.
Secondly, her most meaningful insight was realizing that each member is human and should be regarded as such without the influence of age, sex, or status. Accepting others as human and being accepted by the group members in return, was experienced as a safe place. She said: “It has been a corrective experience for me, for the first time in my life I am not afraid of people.” She added while looking at the two drawings: “I am among friends here, we hug and support one another. I feel so close to you all, as I never had in my entire life.” It should be reiterated that although the second figure is not drawn locked in a bubble as the first, the fact that there are no feet shown, means she still is metaphorically not on a safe ground and still in the process of becoming more “grounded.”
Example 3
This third example is 43 year old female digital media lecturer who lives with her parents (see Figure 3). She shared with the group her sadness of being away from her family when her grandfather whom she loved died, and the fact that her mother had cancer. The secretive and general non-communicative nature of her family prevented her from being able to share her feelings. She felt invisible and unimportant in the family while at the same time expected to bring respect to it by obtaining an education and a high status position.

Being pushed away from the meaningful experiences occurring in her family was internalized by her as betrayal tinged with exploitation. In her first figure she drew herself as a rectangular bottle, with the torso as the bottle and the head as the cap. The averted eyes indicate her anxiety. The boxy shape of the torso represents a protective stance and her case of a shield from depression.

When referring to the activities, she mentioned the “guided imagery with music” session as meaningful, as it assisted her delving into herself and sorting out her feelings, setting the stage to share them with others. Then in regard to the second drawing she said: “Allowing myself to express my negative feelings felt like an internal cleaning. My feelings and attitudes toward my parents has changed. I started talking to them about myself and our relationship—and most surprisingly they are responding in kindness.” The change in her attitude is reflected in her second figure drawing. Her eyes are looking straight ahead, she is smiling and the figure stands on her feet with her arms and hands to the side ready to connect to and interact with her environment.

### Summary of the results

Participants have expressed in the semi-structured questionnaire growth and better well-being. The impression of the figures drawn at the end of the program and their posture seemed to support this feeling of growth.

**Figure 4:**
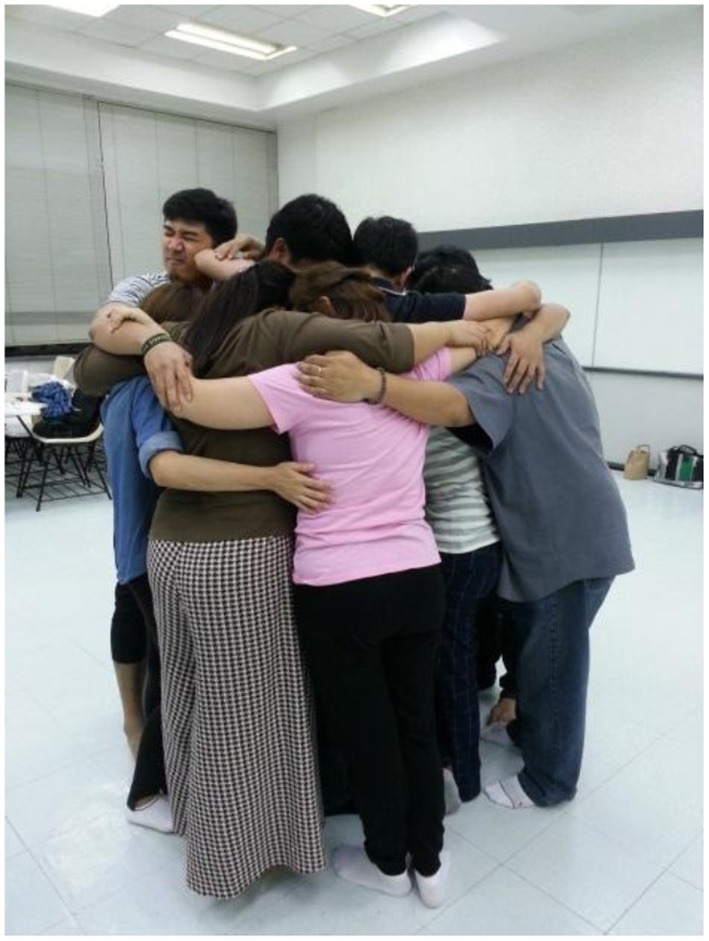
The “comfort-table” hug at the end of the course.

An association between narratives given to the drawings were also exhibited in the verbal report given in the semi-structured questionnaire; for example, impression of the drawn face and feeling at the end of the program, change in their perception of their role as lecturers and face impression at the end and body posture (see Figure 4). The two study measures seemed to promote validation of the reported growth. This can be seen by the group's ending photo picture taken by the lecturer assistant. It shows the farewell hug and entitled the group comfort-table.

## Discussion

This study sets forth the perceived impact of experiential learning on students' personal and professional growth. The “Introduction to Expressive Art Therapies” course presented here, aimed to provide the theoretical background, knowledge, and experience in the use of creative art means for therapeutic and diagnostic purposes. The results indicate that despite the students' initial expectations from the course (which was to broaden their knowledge in expressive art therapy through listening only), the experiential learning element within the course contributed to their personal well-being, improvements in their family and spousal relationships, enhanced social skills, as well as a changed self-perception in roles as lecturers and therapists. These results are consistent with Ko (2014), who reported an increased sense of happiness and optimism, and a better understanding of the therapist's role after completing a movement-based supervisory course.

Not surprisingly, the use of art means was found to be a significant factor in the student's experience and growth. During artmaking, ideas lead to the creation of imagery which in turn generates knowledge, more thought, then more imagery, and so on (Marshall, 2007). In this form of experiential learning, there is a connection between first manipulating materials to create an art piece and then making sense of it (Hickman, 2007). Marshall (2007) asserted that artmaking allows information to be seen differently, in a fresh, more meaningful, personal, and experiential way (as in art, symbolism, and metaphor). This transformation of concepts through imaging, followed by reflection, produces insights and learning. The actual involvement of the person in the interpretative process (through reflection) could utilize the drawing or any other art means, as a prompt or reminder. For instance, in discussing sensitive or difficult issues such as depression or suicidal thoughts (as described earlier in Figure 1). This is in line with previous findings (Wasserman and Beyerlein, 2007) showing that self-figure drawings are effective tools for enhancing self-reflection (a process that involves playing back a period of time related to previous valued experiences in search of significant discoveries or insights about oneself), and self-assessment (a process used for studying one's own performance in order to improve it), two meaningful processes that can lead to learning from experience Reflection is.

The in-class learning process also encouraged group interaction, here-and-now responses, real-time associations, and inspired conceptualization and understanding theoretical models such as post-traumatic growth and group-as-a-whole theory. Students refer both to their fellow students and to the group as meaningful resources supportive of new learning experiences; they also referred from an emotional personal level and cognitive understanding of professional terms such as intimacy, trust, ability to contain, and growth. The model's premise is that the case material presented in the class would stimulate parallel “material” in students, which was then used to elucidate what had taken place in therapy group. Thus, it seems that the findings are more than just the personal and educator's growth, but also incorporated theoretical learning of what is the meaning of therapeutic process.

The employment of experiential learning within academic therapy programs is intended to bridge the theory-practice knowledge gap which in turn may instill new knowledge. While exceptional academic supervisors and mentors go to great lengths to assist students making the connections between study and work, university work-related programs tend to lack the frameworks and support mechanisms bridging theory and practice. As a consequence, many academic supervisors are unwilling or unable to provide the facilitative learning students need before, during, and after their work life placements (Walker, 2004).

## Study limitations and conclusions

Despite the limitations of this study with its small sample size and the fact that all students had previous art education that may account for their ease with art means and the outcomes of this study, it seems that the experiential learning experiences through artmaking reinforced the course content and the presented theories. Students learned through student-centered rather than instructor-centered experiences by doing, discovering, reflecting and applying, and conceptualizing independently and as a group. Through these experiences, students developed communication skills, strengthened self-confidence and decision-making skills by responding to and solving real problems and processes. The acquisition of new unaccustomed modes of behaviors such as touching one another, hugging, sharing and disclosing private personal issues, were indicative of change by the participants which was beyond their existing cultural boundaries. Thais are not used to touching or hugging friends or colleagues and often the same can be said about family members. They also tend to avoid burdening others with their own personal difficulties and remain silent to the point of refraining from asking questions in class to avoid any potential embarrassment of the lecturer out of respect.

In conclusion, the findings of this study can serve as a foundation for developing an effective, flexible, creative, and culturally appropriate model of an experiential learning element within academic therapy programs in general and in expressive arts therapy in particular. Experiential learning as described in this study involves the whole person (all aspects of human experience- physical, mental, emotional, and intellectual). This form of learning takes place along the affective, cognitive and behavioral dimensions since it consists of participative, interactive, and applied elements. It also allows contact with the environment, and exposure to processes that are highly variable and uncertain. Obviously, the experience needs to be structured to some degree and the relevant learning objectives need to be specified along with monitoring of the experience to maintain the course's objectives (Gentry, 1990). It is important to note that students need to evaluate their experience in light of relevant theories and in light of their own feelings. Moreover process feedback needs to be provided to the student to complement (and possibly supersede) the outcome feedback received by the student. Nevertheless, further research focusing on experiential learning's influence on the therapeutic skills and knowledge, is needed in order to develop better exercises and improve academic therapy programs.

## Ethics statement

This study was carried out in accordance with the recommendations of CU Ethical Board Committee. The protocol was approved by the CU Ethical Board Committee.

## Author contributions

BB was in charge of the background, course program, and discussion. RL-W was in charge of the methodology and results.

### Conflict of interest statement

The authors declare that the research was conducted in the absence of any commercial or financial relationships that could be construed as a potential conflict of interest.
